# A resonant cavity system for exposing cell cultures to intense pulsed RF fields

**DOI:** 10.1038/s41598-022-08662-7

**Published:** 2022-03-19

**Authors:** Masood Ur-Rehman, Yasir Alfadhl, Xiaodong Chen, Rachel Whiting, Alex Wright, Christopher D. Lindsay, John Tattersall, Iain Scott

**Affiliations:** 1 James Watt School of Engineering, University of Glasgow, University Avenue, Glasgow, G12 8QQ Scotland UK; 2 School of Electronic Engineering and Computer Science, Queen Mary University of London, Mile End Road, London, E1 4NS England UK; 3 Chemical, Biological and Radiological Sciences Division, Defence Science and Technology Laboratory (Dstl), Porton Down, Salisbury, SP4 0JQ Wiltshire UK

**Keywords:** Electrical and electronic engineering, Biophysics, Cell biology

## Abstract

The IEEE and ICNIRP had specified a maximum permissible exposure for instantaneous peak electric field of 100 kV/m. However, no rationale was given for this limit. A novel exposure system was designed through a detailed process of analytical analysis, numerical modelling and prototype testing. The system consists of a cylindrical re-entrant resonant cavity that can achieve an electric field strength of more than 100 kV/m with an input power of 200 W. The working of the system was evaluated in simulation and experiment in terms of scattering parameters, electric field distributions and specific absorption rate. The system was then used to carry out in-vitro exposures of a human lymphoid cell line (GG0257) to a 1195 MHz signal at 53 dBm peak power and a pulse width of 550 ns at a range of interpulse intervals to identify heating-induced changes in cell viability. The proposed system offers high Q value of 5920 in unloaded condition which was reduced to 57 when loaded with 12 ml of cell culture but still offering 67 kV/m of the field intensity. Using the system for the exposure of GG0257 cells lasting 18 min, interpulse intervals of 11 μs or less caused a reduction in the number of viable cells and a corresponding increase in necrotic cells. For a shorter exposure duration of 6 min, the reduction in cell viability was seen at interpulse intervals of 5.5 μs or less. The designed exposure system is well capable of handling high intensity electric fields. Temperature measurements with a fibre optic probe and temperature sensitive labels showed that changes in viability were associated with temperature increases above 46 °C. This novel exposure system is an efficient means to investigate the possible relationship between peak field intensity and biological effects to provide a rationale behind the maximum exposure limit of 100 kV/m.

## Introduction

Science-based guidelines for exposure to electromagnetic (EM) fields, such as those published by ICNIRP^1^ and IEEE^2^, are based on the established effects of these fields on biological systems. For radio frequency EM fields, the established biological effect is tissue heating and the guidelines are intended to limit temperature rises induced by time-averaged heating^3^.

Earlier versions of both the guidelines specified limits on peak field intensity for pulses. The 2005 IEEE standard^2^ specified a maximum permissible exposure for the instantaneous peak electric field of 100 kV/m. No rationale was given in the 2005 standard for this limit, but an earlier version of the standard^4^ stated that:

“The recommendation for a peak E-field limit of 100 kV/m is based on the necessity to cap the allowable field below levels at which air breakdown or spark discharges occur.”

Similarly, the ICNIRP 1998 guidelines suggested that, for frequencies exceeding 10 MHz, equivalent plane wave power density as averaged over the pulse width should not exceed 1000 times the reference levels or that field strengths should not exceed 32 times the field strength reference levels^5^, while recognising that:

“Little information is available on the relationship between biological effects and peak values of pulsed fields.”

Thus, although limits were specified for peak field intensity, these were not based on any known biological effects and were removed from the latest version^1^.

Though a wide range of low power exposure systems to study the biological effects of EM fields emitted from microwave equipment have been described in the literature^6–18^, systems that can handle very high electric fields are relatively scarce. Moreover, these exposure studies have struggled from the issues of re-creation, reproducibility, and reliability^19^. In-vitro exposure studies also require a uniform field distribution to ensure that all the cells are exposed at a similar Specific Absorption Rate (SAR). A further requirement is for the system to fit inside a cell culture incubator.

Classical closed exposure systems capable of handing high electric fields can be divided into three categories^19^; (1) TEM cell exposure systems; (2) rectangular waveguide exposure systems; and (3) resonant cavity exposure systems. A typical TEM cell is a rectangular coaxial transmission line tapered at each end forming a metal shielded environment establishing the Transverse Electro-Magnetic (TEM) mode of EM field over a given range of frequencies. The TEM type of cell exposure system has long been used in exposure studies since its invention by Crawford^7,11,20–23^. TEM cells provide exposure conditions closest to free-space environment. Although, the electric and magnetic fields are well characterised, maintaining temperature in TEM cells is not easy and a thermal gradient always exists. Therefore, they are not a preferred choice for high power exposures.

Rectangular waveguides are another popular category of exposure systems. They confine the power inside the apparatus due to the total reflection of the EM wave from the waveguide walls^16,24–30^. However, these systems are bulky and hard to fit inside a cell culture incubator, making the environmental control difficult. Moreover, they have a narrow operating frequency range and the limited interior size of the waveguide’s exposure chamber restricts the usage to specific types of cell culture containers^19^.

Resonant cavity exposure systems have also been used widely in the study of biological effects on cell cultures. They offer high volume efficiency and compact size, enabling easy placement in the incubators^19^. The electrical parameters of resonant frequency, quality (Q) factor and impedance matching are highly sensitive and therefore, very challenging to achieve^11^. A well-matched resonant structure absorbs all of the incident power increasing the efficiency and having a capability of high electric field magnitude^31^. A number of resonant systems for exposing cell cultures have been presented in the literature. Although, the majority of these resonant cavity exposure systems employ rectangular waveguides shortened at one end^32–38^, a cylindrical structured resonant system can irradiate the enclosed sample with highly uniformly distributed field across the container^39–43^. Cylindrical resonant cavities have also been designed for in-vivo tooth dosimetry^44^, enzymatic homogeneous hydrolysis of sucrose^45^, dielectric measurements of common solvents^46^, and electron paramagnetic resonance spectroscopy^47^. Re-entrant cavity resonators further improve these characteristics and have been used in many applications including particle accelerator, electron spin spectroscopy, dielectric characterization, microwave oscillator, displacement sensor, tunable resonators, and filters^43,48^.

As recognised by ICNIRP, there have been relatively few studies on the possible relationship between peak field intensity and biological effects. This study is an effort to tackle this important issue. The literature shows that TEM cells are not preferred for high power exposures, while the rectangular waveguides do not fit for the purpose of this study due to the size and power handling constraints^49^ introduced by the available hardware (Agilent E4432B signal generator and the incubator). A resonant cavity appears to be a good instrument to study these effects offering a simple geometry, high Q levels and a uniform field pattern across the sample container.

This work has developed a novel system based on a cylindrical re-entrant resonant cavity that can achieve high electric field strength for the exposure of cell cultures. The system supports field strength of more than 100 kV/m with an input power of 200 W. In unloaded conditions, this system exhibits good impedance matching at 1800 MHz, with a Q factor of 5920. Loading the cavity with a 55 mm diameter petri dish containing cell culture shifted the resonant frequency to 1195 MHz and reduced the Q factor to 57. A detailed numerical analysis followed by the reflection coefficient measurements was carried out to validate the working of the exposure system. A human lymphoid cell line (GG0257) was then used to identify exposure thresholds for heating-induced biological effects using the designed exposure system, which is a necessary requirement before searching for effects of peak field alone (i.e. no heating). The duty cycle was decreased (by increasing the interpulse interval) whilst maintaining a constant peak field, until the absorbed energy was insufficient to cause heating-induced decreases in cell viability. Future experiments will use duty cycles below this threshold in order to search for effects which may be specifically related to peak field intensity. The results of this work will provide evidence for assessing the requirement for peak field limits in exposure guidelines for pulses.

## Materials and methods

**Figure 1:**
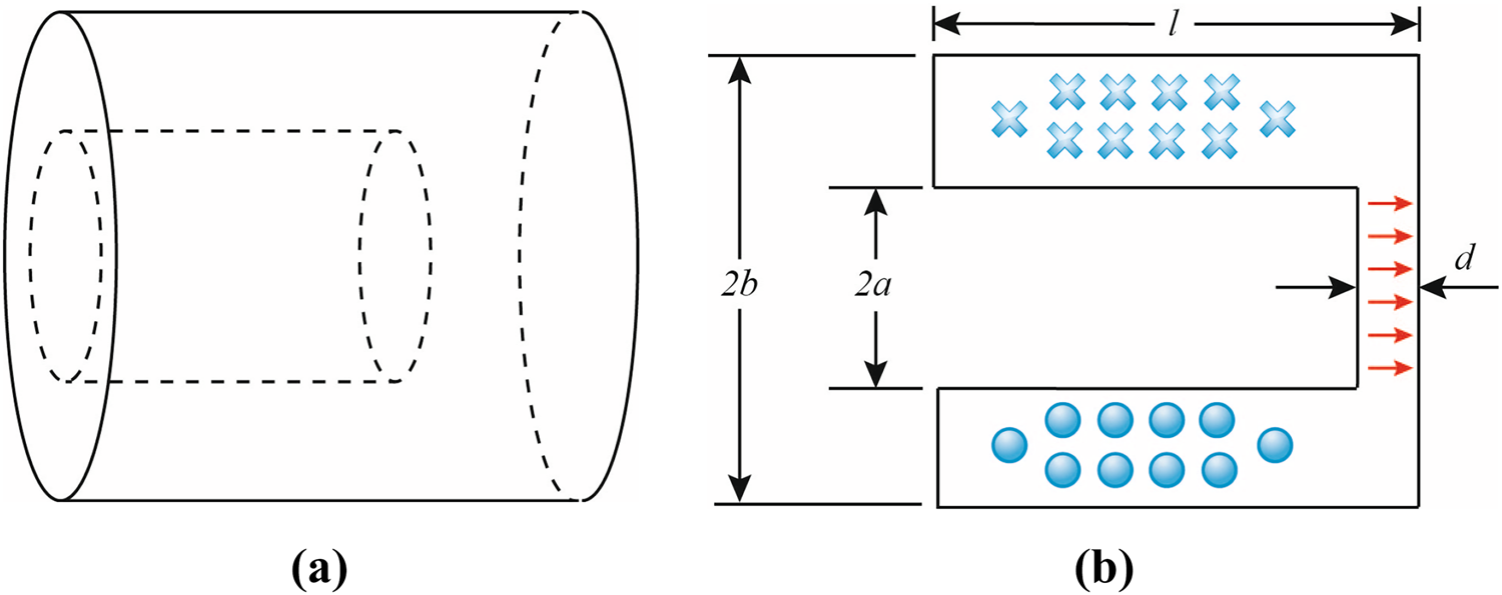
A cylindrical re-entrant cavity resonator with key structural dimensions; (**a**) Cavity structure; (**b**) Key dimensions and field configuration for $TE_{111}$ mode (crosses: inward H-field, circles: outward H-field, arrows: E-field).

Cylindrical re-entrant cavity structures can be used to excite electric fields in different modes. A re-entrant cavity consists of a short metallic section with a gap in the centre conductor. Re-entrant refers to the extension of the metallic boundaries into the interior of the cavity, as shown in Fig. 1.

The re-entrant structure confines the electric field in a small region increasing its strength significantly. It also retains a high Q factor ($\sim$3000) as the magnitude of the magnetic field is small and spread over a much larger volume resulting in low surface losses. Thus, the re-entrant cavities have the advantage of simple mechanical construction and tuning ability over a wide frequency range while having an overall small size. Moreover, these cavities are highly sensitive to detect even minute changes in the field strength and offer reasonably uniform electric field distribution over the exposed sample. They are widely used in klystrons, solid-state devices and dielectric measurements and these advantages lead us to consider them for the cell exposure to very high RF fields (i.e. >100 kV/m strength) in this study.

### Analytical design

The key parameters that define the performance of a re-entrant cavity include length of the cavity ($l$), gap between the re-entrant stage and cavity wall ($d$), radius of re-entrant stage ($a$) and radius of the cylinder ($b$). Different techniques can be used to feed the cavity of which loop structures are most widely adopted.

The fundamental mode ($TE_{111}$) frequency of the radial cavity shown in Fig. 1 can be calculated using the following expression^48,50^:

1 $$\begin{aligned} f= & {} \frac{c}{2 \pi \sqrt{\epsilon _r} } \left[ al \left( \frac{a}{2d} - \frac{2}{l} \ln {\frac{0.765}{\sqrt{ l^{2} + {\left( b-a \right) }^2 } } } \right) \ln \frac{b}{a} \right] ^{-\frac{1}{2} } \end{aligned}$$

where $c = 3 \times 10^8$ m/s is the velocity of light and $\epsilon _r$ is 1 for the air-filled cavity. There can be infinite number of modes in such cavities. The field distribution for $TE_{111}$ mode is illustrated in Fig. 1b. In this mode, the electric field is concentrated in the gap region making high electric field strength achievable. Therefore, $TE_{111}$ mode is chosen as the dominant mode in the proposed cavity design.

Field strength in the cavity resonators depends on the Q factor calculated as^51–53^:

2 $$\begin{aligned} {Q}_c=\frac{ {2{\omega }_o} {W}_e}{{P}_c} \end{aligned}$$

where, $W_{e}$ is the stored electric energy while ${P}_c$ is the power dissipated in the cavity walls. The operating frequency is given by ${\omega }_o$.

### Numerical modelling

The in-vitro exposure system design has to follow a set of requirements leveraged by the available test equipment. These requirements are as follows:

- It should achieve a peak electric field intensity of 100 kV/m with 200 W of input power.
- The resonance frequency in unloaded and loaded conditions should lie between 1000 and 2000 MHz, a limitation imposed by the available power source (Agilent E4432B signal generator).
- The system should be compact with the largest dimension not more than 150 mm to fit in the incubator.
- The system’s dimensions should be large enough to accommodate a 55 mm petri dish containing the cell culture while providing a stable platform for the sample and a uniform field distribution across the container.

The numerical design was carried out through a laborious optimisation process as the individual dimensions for the required performance cannot be calculated directly. Equation (1) governs the choice of initial cavity dimensions. CST Studio Suite software that uses Finite Integration Technique (FIT)^54^ was used to model the proposed exposure system and analyse its performance numerically. A coupled loop structure was used to excite the $TE_{111}$ mode in the cavity as it has a relatively wider reactive matching range. The reflection coefficient, electric field and Q factor were employed as the performance metrics.

#### Unloaded cavity

**Figure 2:**
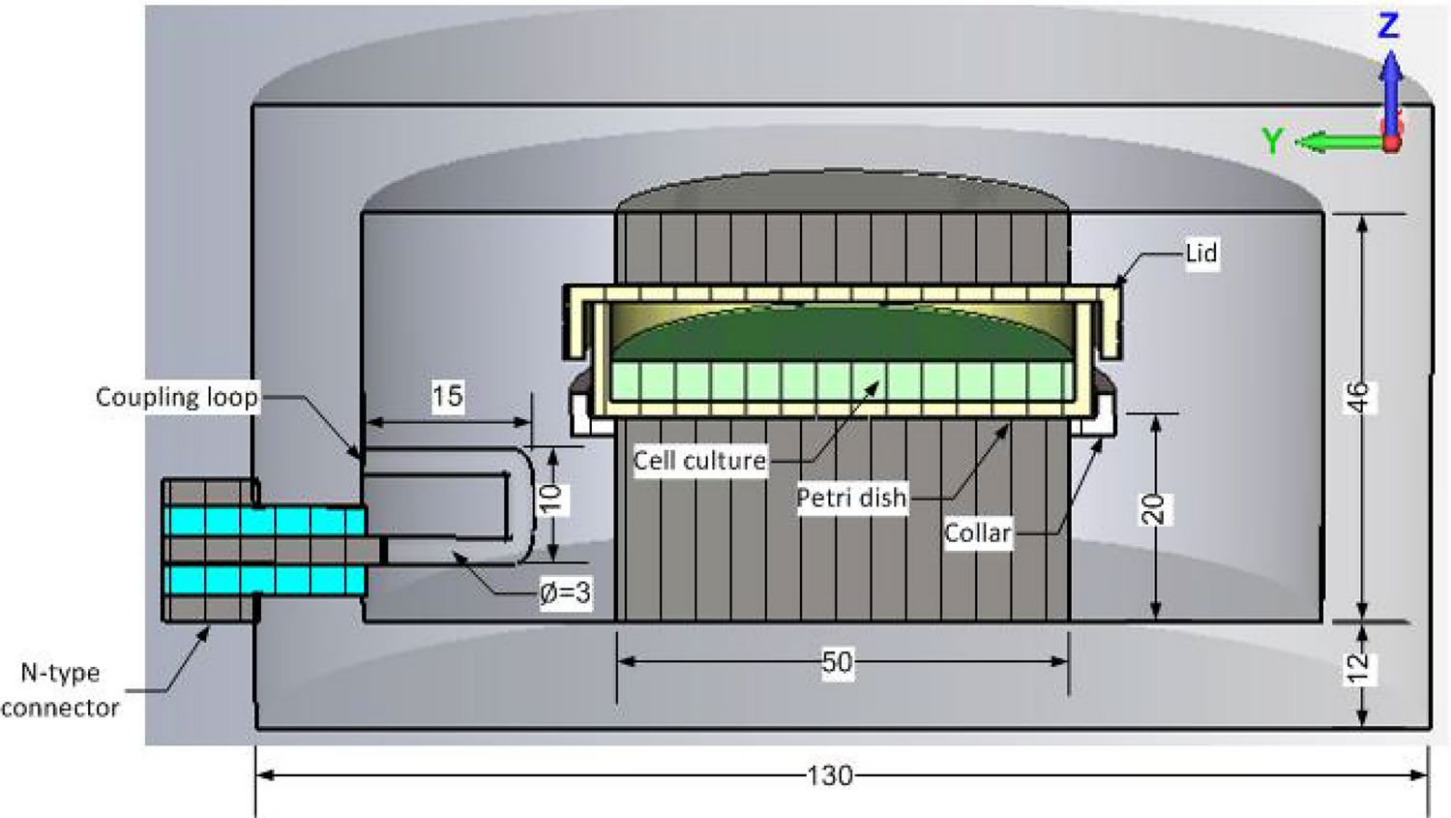
Schematic layout of the initial design of proposed cylindrical re-entrant cavity exposure system (all lengths are in mm) showing cross-section view of loaded cavity with 12 ml cell culture contained in the petri dish.

Numerical model of the initial design of the proposed cylindrical re-entrant cavity along with the structural dimensions is given in Fig. 2. Power was fed to the system using an N-type coaxial connector. A PTFE collar with dielectric constant of 2.1 was included to hold the petri dish and stabilise the cell culture. To increase the field strength inside the cavity and enhance mechanical stability, a dual re-entry arrangement was utilised. The gap between the two re-entry stages was kept at 16.2 mm to provide enough room for the petri dish with lid.

**Figure 3:**
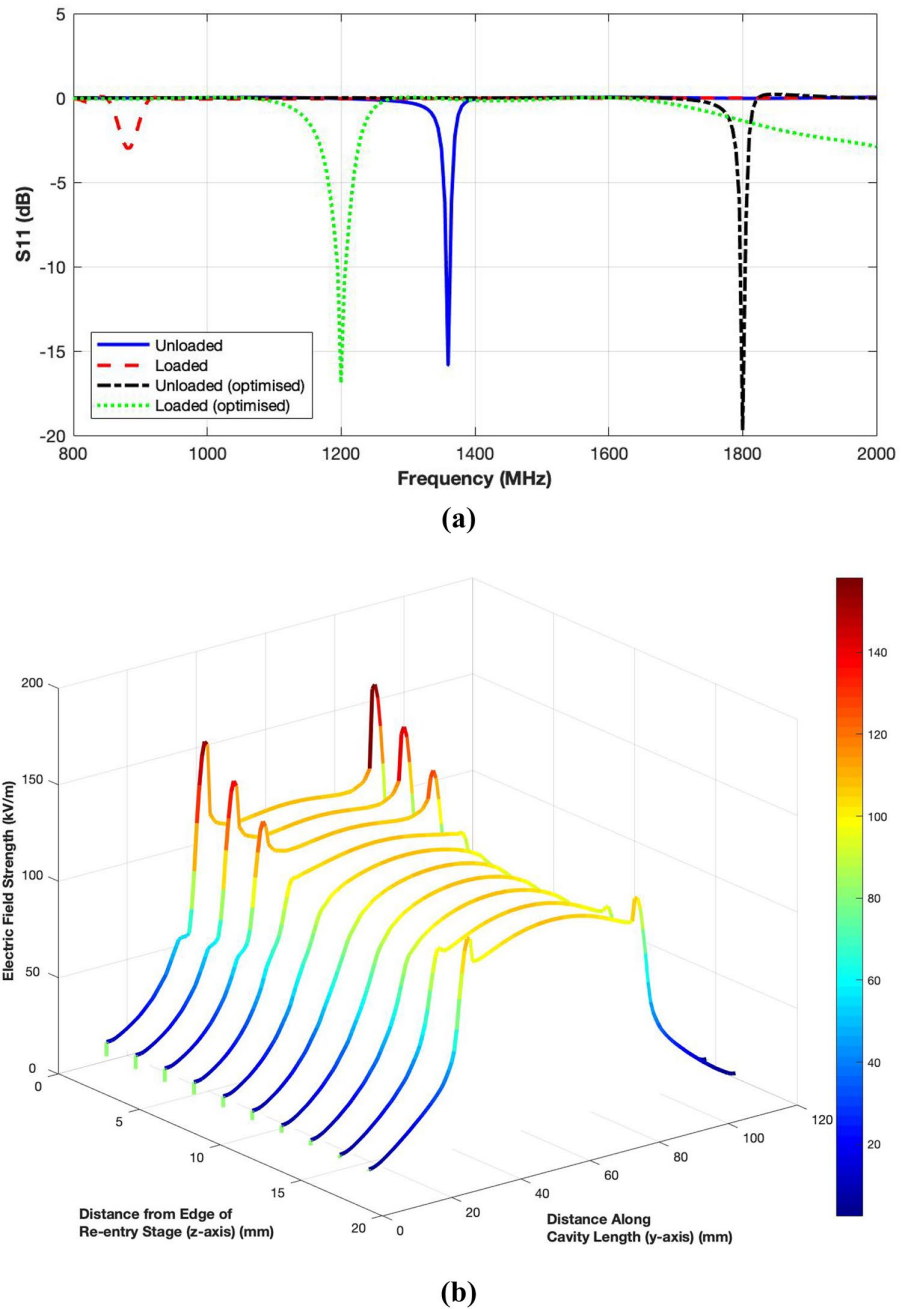
Performance analysis of the proposed exposure system; (**a**) Reflection coefficient response in unloaded and loaded conditions; (**b**) Electric field profile on curves spanning the cavity length and width in unloaded condition.

The performance of the proposed exposure system was first analysed in terms of reflection coefficient ($S_{11}$) response shown in Fig. 3a. A strong resonance was observed at 1350 MHz when the cavity was unloaded. The magnitude of the reflection coefficient was noted to be −16 dB that indicates a very good matching. Figure 3b presents electric field distribution viewed on the centre face of the cavity calculated on 10 curves covering whole of the gap region between the two re-entry stages.

The electric field distribution shows that the system can achieve a peak field strength of 120 kV/m. Also, the field profile remains largely uniform along the length of the cavity. The high peaks indicate the metal edges of the larger re-entry stage. The Q factor for the proposed system was noted to be 9533. These results show that the design of cylindrical re-entrant resonant cavity exposure system efficiently meets all the design requirements.

#### Loaded cavity

The resonant cavity is intended to be used to expose the human tissue cells to a very high electric field making it pertinent to evaluate its performance in loading conditions. A standard polystyrene petri dish of 55 mm diameter, 14.2 mm height and $\epsilon _{r}$=2.4 has been designed to contain the cell culture for the exposure studies^55,56^. These petri dishes are the optimised choice sanctioned by the size of the incubator and restricted space available within the cavity chamber. Using a larger petri dish would require larger cavity and affect its ability to fit into the incubators available while a smaller petri dish cannot be held mechanically stabilised. Use of more than one petri dishes is not feasible again due to the system’s form factor. Moreover, it would have had performance implications with respect to reflection responses and electric field distribution profiles.

A 12 ml cell culture medium (RPMI 1640, Roswell Park Memorial Institute Medium) with relative permittivity, $\epsilon _{r}$=66.14 and conductivity, $\sigma$=1.81 S/m (based on measurements at 1275 MHz and 37 °C) having mass density, $\rho$=1050 kg/m$^3$ contained within the 55-mm petri dish was considered to simulate and replicate the worst case loading scenario.

The geometry of the loaded cavity is illustrated in Fig. 2. The reflection coefficient result given in Fig. 3a shows that loading the cavity with 12 ml cell culture in the petri dish severely affected the performance by introducing impedance mismatch and a resonance shift to 883 MHz.

### Optimisation of exposure system

Since, loading the exposure system with the cell culture moves its resonant frequency out of the band of interest (i.e. 1000–2000 MHz), the cavity design needs to be optimised. As loading the cavity with petri dish and cell culture introduces a capacitive effect, an inductive loading of the system can effectively balance it out and minimising the frequency detuning.

**Figure 4:**
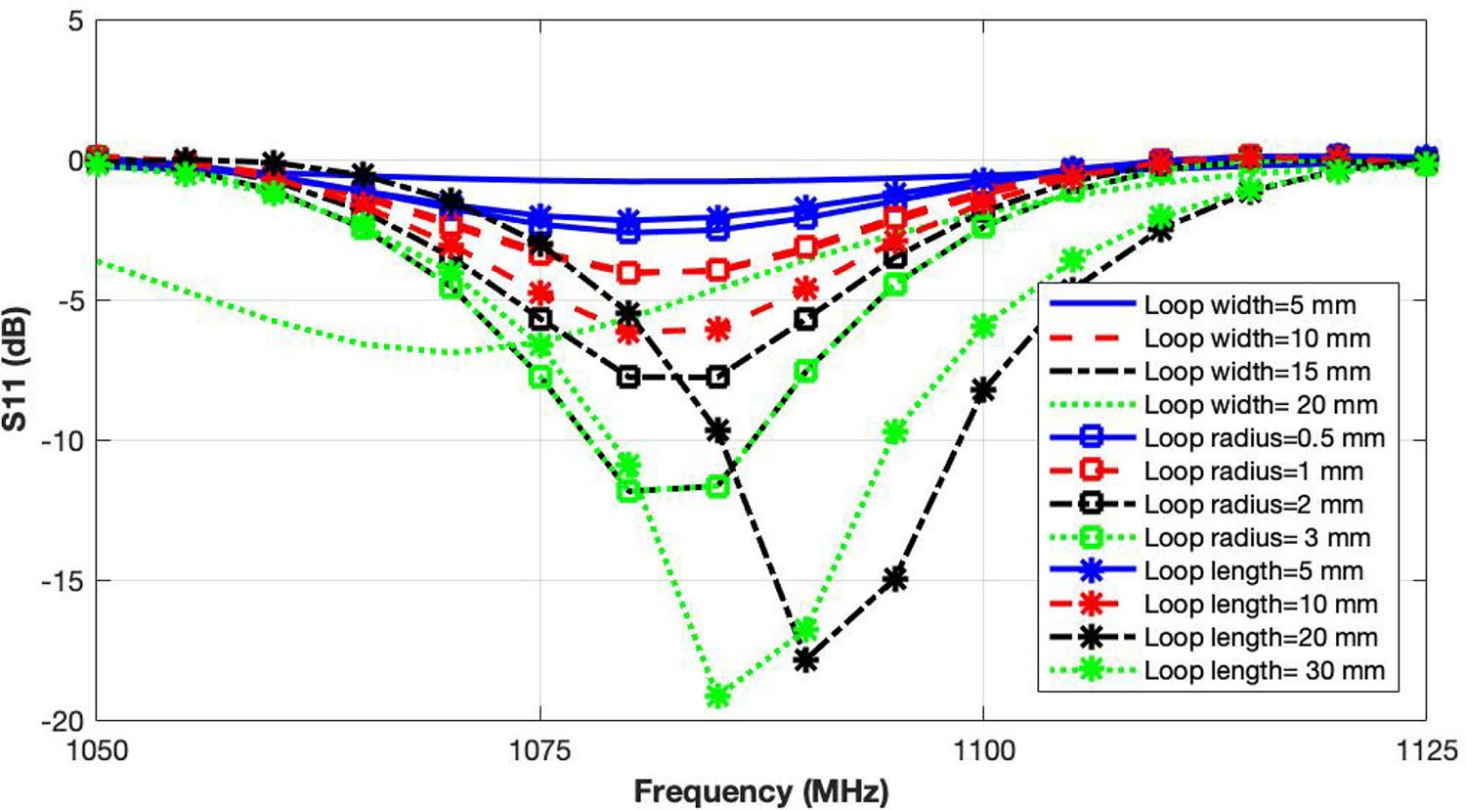
Re-tuning the loaded cavity exposure system by changing coupling loop dimensions.

Two balancing techniques were applied to re-tune the loaded cavity within the band of interest; (1) varying coupling loop dimensions; and (2) reducing the empty space inside the cavity. Varying the dimensions of the coupling loop can change the impedance matching significantly. Different variations in the loop structure were investigated in simulation to see the effects on the reflection coefficient. The three varied parameters are radius, length and width of the coupling loop. The reflection coefficient curves for these variations are compared in Fig. 4. A significant effect of changing the coupling loop dimensions on the impedance matching can be observed. Though the dimensions of all three structural parameters of the loop affect the impedance matching, adopting a largely changed single parameter would not work due to limited inner space of the cavity. Hence, the three parameters need to have a carefully optimised increment for better impedance matching. A loop with dimensions of 30 mm length, 15 mm width and 2 mm radius was therefore, adopted based on this optimisation study.

**Figure 5:**
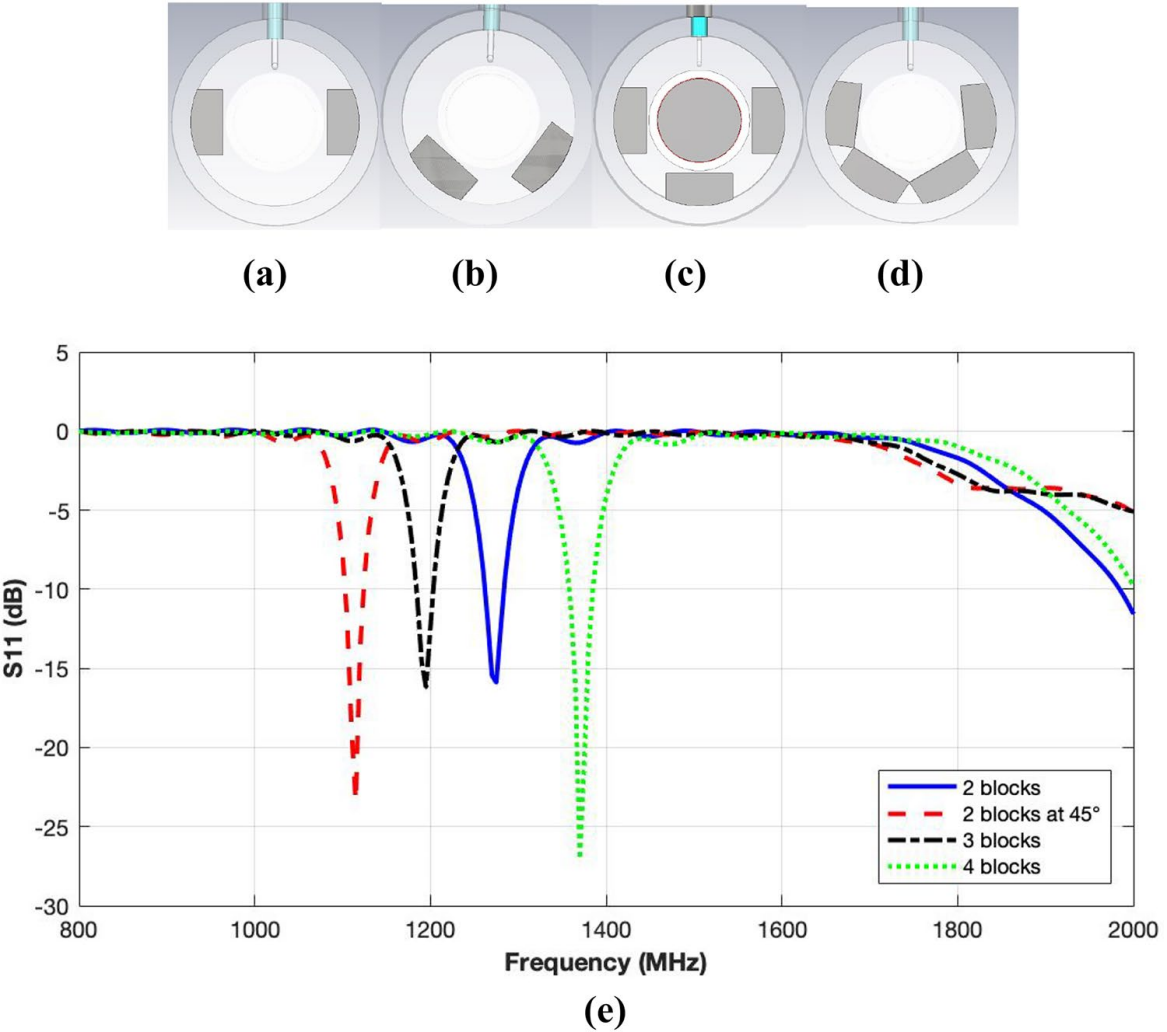
Geometry of loaded cylindrical re-entrant resonant cavity with metallic blocks; (**a**) Two blocks; (**b**) Two blocks at 45°; (**c**) Three blocks; (**d**) Four blocks; (**e**) Reflection coefficient responses for loaded cavity with metallic blocks.

The second method involves reduction in the cavity’s inner space. Metallic blocks, 2 to 4 in number and placed at different angles with respect to the cavity’s vertical axis, were considered as shown in Fig. 5a–d. Figure 5e compares the reflection coefficient performance of the cavity with varying number of the blocks and excited using the larger coupling loop. The results exhibit that inclusion of the metallic blocks do improve the impedance matching and retuning of the cavity through an upward resonance shift. Greater the number of the blocks, the larger is the frequency shift with inclusion of four blocks giving the best performance. Since, the four blocks are touching each other at the front end, it is expected that a semi-circular metallic ring of horse-shoe shape would perform even better.

#### Optimised unloaded cavity

Study of the impedance matching techniques revealed that the loaded cavity works well when a larger excitation loop along with a semi-circular metallic ring for inductive loading is used. Based on these findings, the cylindrical cavity resonator design was updated. The numerical model was simulated and results were analysed.

Although, the larger excitation loop works well for the loaded cavity, it falls short to provide the same impedance matching for the unloaded scenario. Hence, the N-type connector is designed to support a changeable coupling loop. The original smaller loop is used for the unloaded cavity and needs to be detached and replaced by a larger loop when the cavity is used for the exposure of the cell culture. Since, the primary usage of the cavity would be in loaded condition, replacing the loop is rarely needed and it is mostly for the confirmation of the simulated results through experiment.

**Figure 6:**
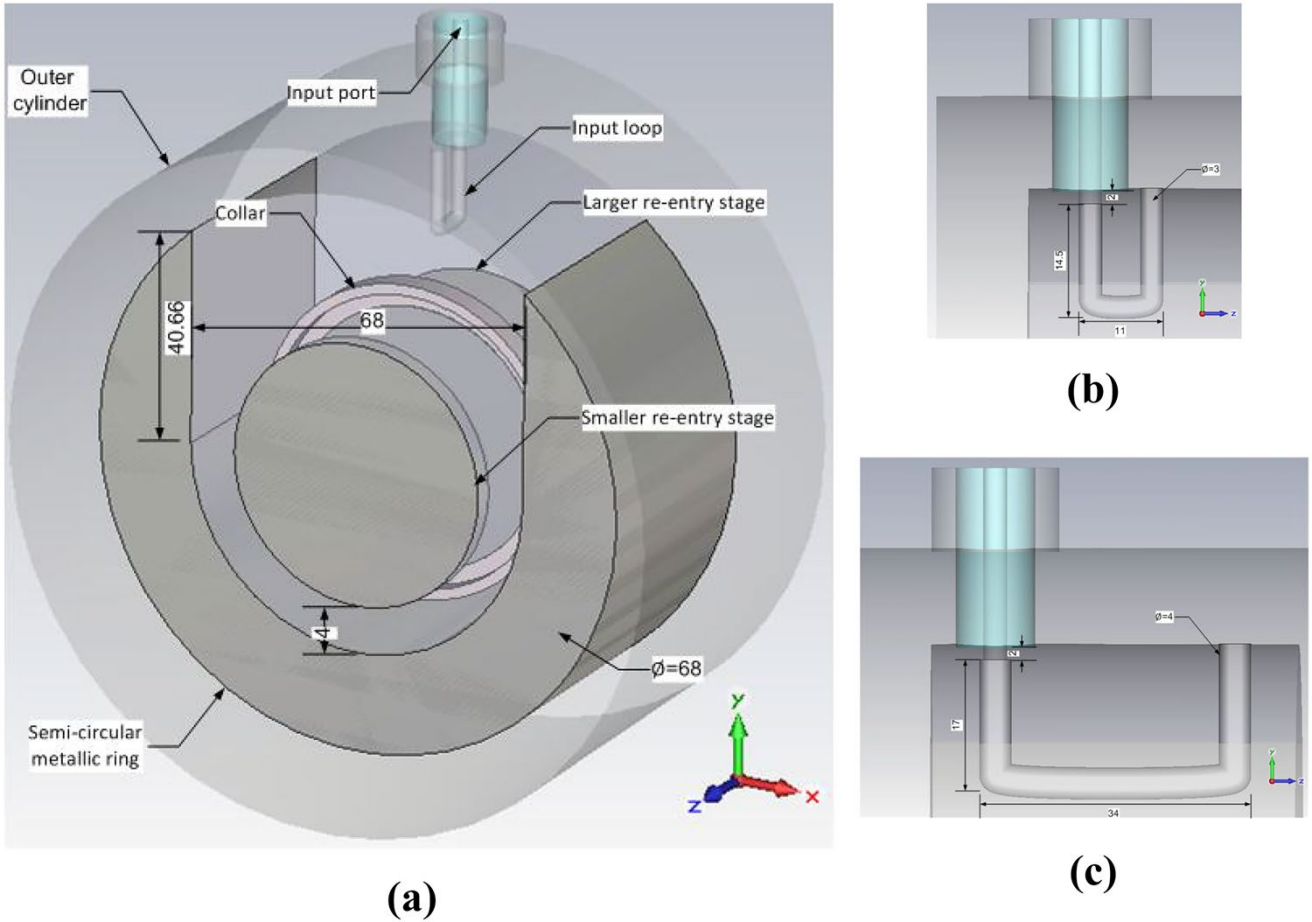
Geometry of optimised cylindrical re-entrant resonant cavity exposure system with a semi-circular metallic ring for impedance matching (all lengths are in mm); (**a**) Components of the cavity; (**b**) Dimensions of smaller loop for the excitation of unloaded cavity; (**c**) Dimensions of larger loop for the excitation of loaded cavity.

**Figure 7:**
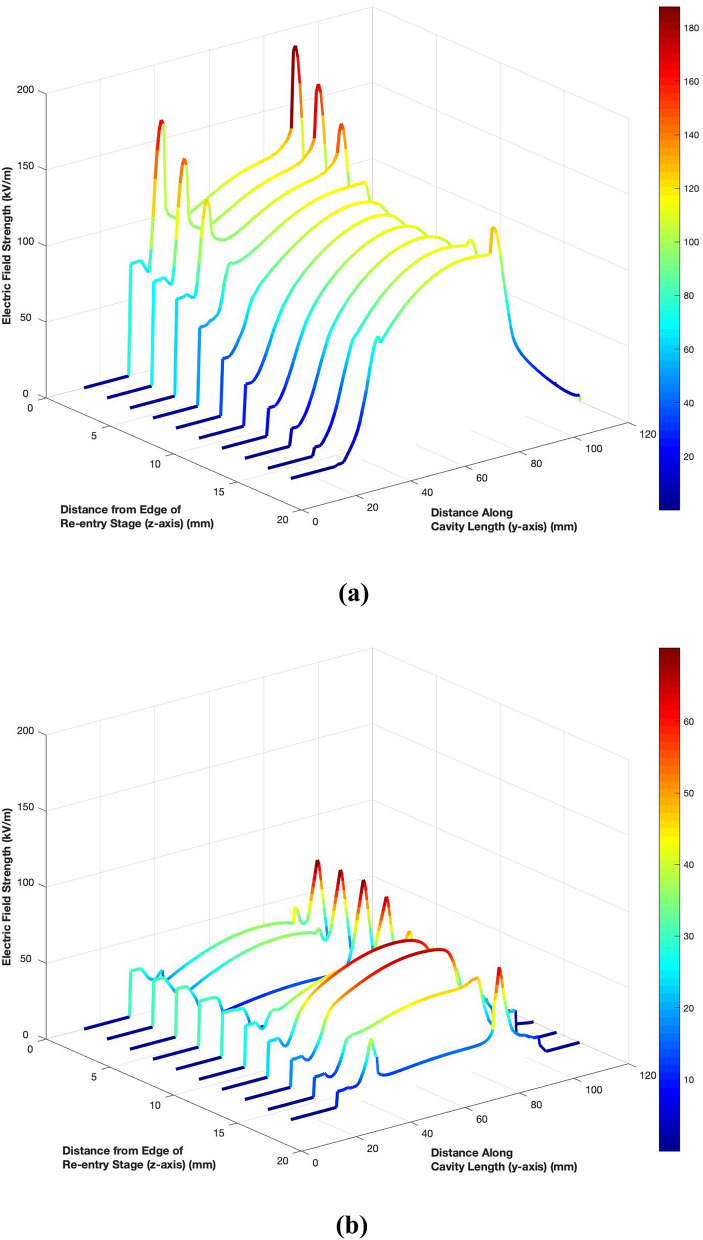
Electric field distribution for optimised cylindrical re-entrant resonant cavity exposure system with a semi-circular metallic ring; (**a**) Unloaded cavity; (**b**) Cavity loaded with 12 ml of cell culture.

Instead of inserting separate four metallic blocks, a single horse-shoe shaped semi-circular metallic ring was designed as the inductive load. The modified cavity design is presented in Fig. 6a. Due to this addition, slight changes in the smaller coupling loop were required. Fig. 6b, c illustrate the optimised geometries of the smaller and larger coupling loops. The performance of the modified unloaded cavity was then analysed in simulation. The reflection coefficient response is given in Fig. 3a while the electric field distribution calculated on 2 mm apart curves spanning the length and width of the gap region between the two re-entry stages of the cavity are exhibited in Fig. 7. The results show that the modified resonant cavity exhibits excellent impedance matching with a strong resonance at 1800 MHz. The electric field distribution indicates that with the introduction of the semi-circular metallic ring, the strength of the electric field has increased to 125 kV/m due to reduced non-metallic area with a uniform pattern in the middle region where the cell culture would be placed. The peaks appearing at lower Y-axis are due to the proximity of the coupling loop. The Q factor has seen a decrease to 5920 indicating increase in losses due to higher volume of the metal surfaces.

#### Optimised loaded cavity

The modified cylindrical resonant cavity with larger coupling loop for excitation was then tested in loading conditions. The cavity was loaded with a 55 mm petri dish containing 12 ml of the cell culture medium. The dimensions of the petri dish and electric properties of the cell culture were as discussed earlier.

The simulated reflection coefficient (Fig. 3a) shows that a very good impedance matching was achieved despite the loading of the petri dish and cell culture. Although, the resonance frequency is shifted to 1195 MHz, it is still well above the lower end of the band of interest (i.e. 1000 MHz). It is evident from Fig. 7b that, although the electric field strength reduces due to energy deposition in the cell culture, the level of reduction is less as compared to the original loaded cavity design. A maximum field strength of 63 kV/m and Q factor of 57 is noted for the optimised cavity design.

The results show that the optimised cylindrical re-entrant resonant cavity exposure system fulfils all the design requirements. It achieves a peak electric field strength of more than 100 kV/m in unloaded condition. Moreover, it efficiently overcomes the problem of impedance mismatch and resonance shift outside the frequencies of interest.

### Numerical exposure study

The optimised cavity model was employed for a detailed numerical exposure study. The effect of varying cell culture volume inside the petri dish on the impedance matching, resonance frequency, electric field strength and SAR distribution were studied via computer simulations. The input power was considered to be 200 W and the cell culture volume was varied from 2 to 22 ml.

**Figure 8:**
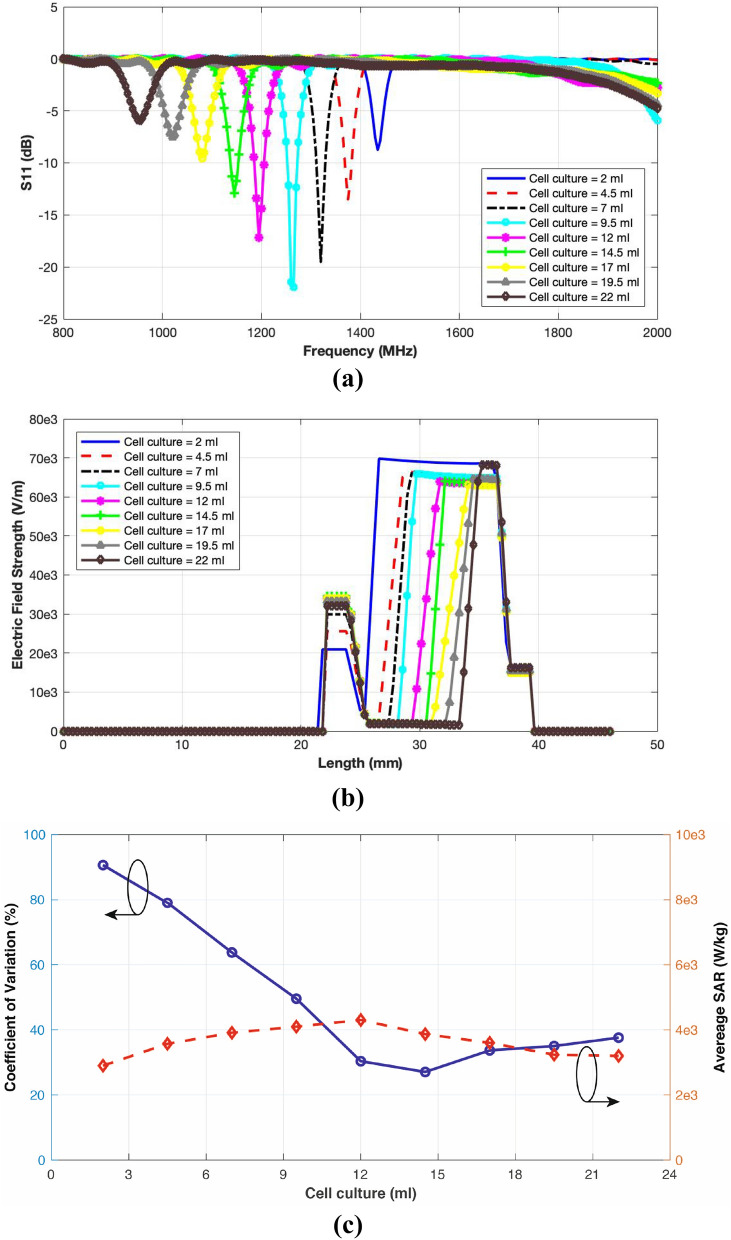
Performance evaluation of the designed exposure system with varying cell culture volume; (**a**) Reflection coefficient response; (**b**) Electric field strength; (**c**) SAR (average values and Coefficient of Variation).

**Figure 9:**
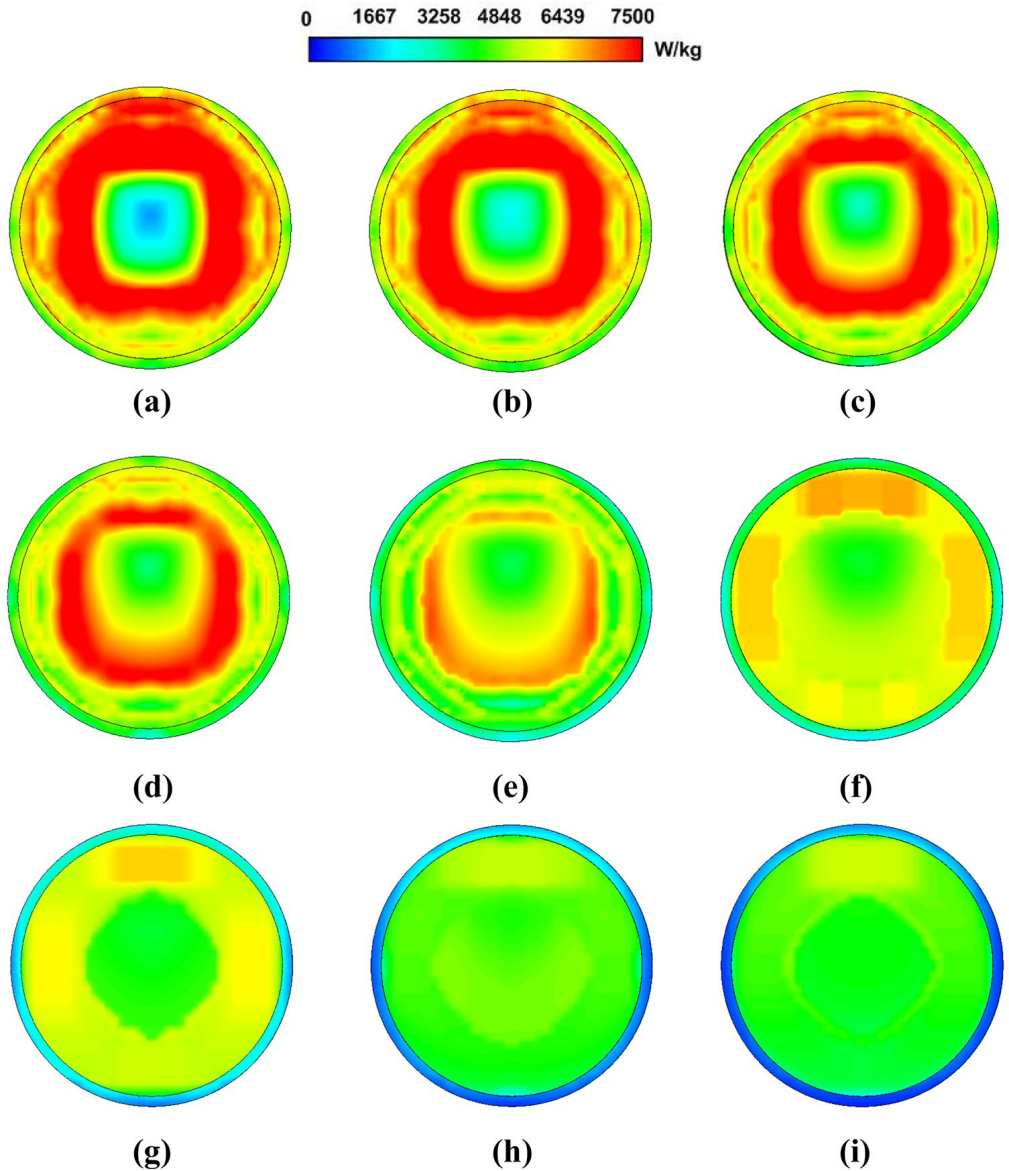
SAR distribution for varying cell culture volume exposures on a horizontal plane in the centre of the cell culture; (**a**) 2 ml; (**b**) 4.5 ml; (**c**) 7 ml; (**d**) 9.5 ml; (**e**) 12 ml; (**f**) 14.5 ml; (**g**) 17 ml; (**h**) 19.5 ml; (**i**) 22 ml.

Figure 8a comparing the simulated $S_{11}$ response for the loaded cavity shows that increase in the cell culture volume brings a downward shift in the resonance frequency in intervals of 50–60 MHz. For 2 ml of the cell culture, the resonance frequency is 1435 MHz that becomes 954 MHz for 22 ml cell culture. It is therefore deduced that the maximum volume should not exceed 19.5 ml to keep the resonance inside the operating bandwidth of the power amplifier. The best impedance matching of −22 dB is observed for a cell culture volume of 9.5 ml with a resonance frequency of 1264 MHz.

The electric field is calculated on the vertical central axis of the cavity and plotted in Figure 8b giving an insight of the field build-up in the presence of the cell culture. It can be observed that increasing the volume of the cell culture decreases the electric field strength due to higher adsorptions. The minimum field strength is observed to be 1.55 kV/m for 22 ml of the cell culture.

The exposure is characterised for different cell culture volumes in terms of average SAR values (calculated using IEEE C95.3 standard^57^) and Coefficient of Variation (CV). CV is a measure of non-uniformity of the SAR values^56^. The results in Fig. 8c reflect a strong dependence of the exposure upon the cell culture volume. Cell culture volume of 12 ml gives the best performance with CV of 30% and average SAR value of 4300 W/kg. Though 14.5 ml cell culture yields least non-uniformity, it has a lower average SAR of 3867 W/kg.

SAR distributions for cell culture exposure in a horizontal cut through the middle of the cell culture medium (Fig. 9) show that the SAR levels are the highest near the petri dish face illuminated by the coupling loop. It then tapers off towards the other end in an annular ring fashion uniformly having minima at the centre of the petri dish. Higher cell culture volumes offer relatively better uniformity.

### Fabrication and testing of exposure system

**Figure 10:**
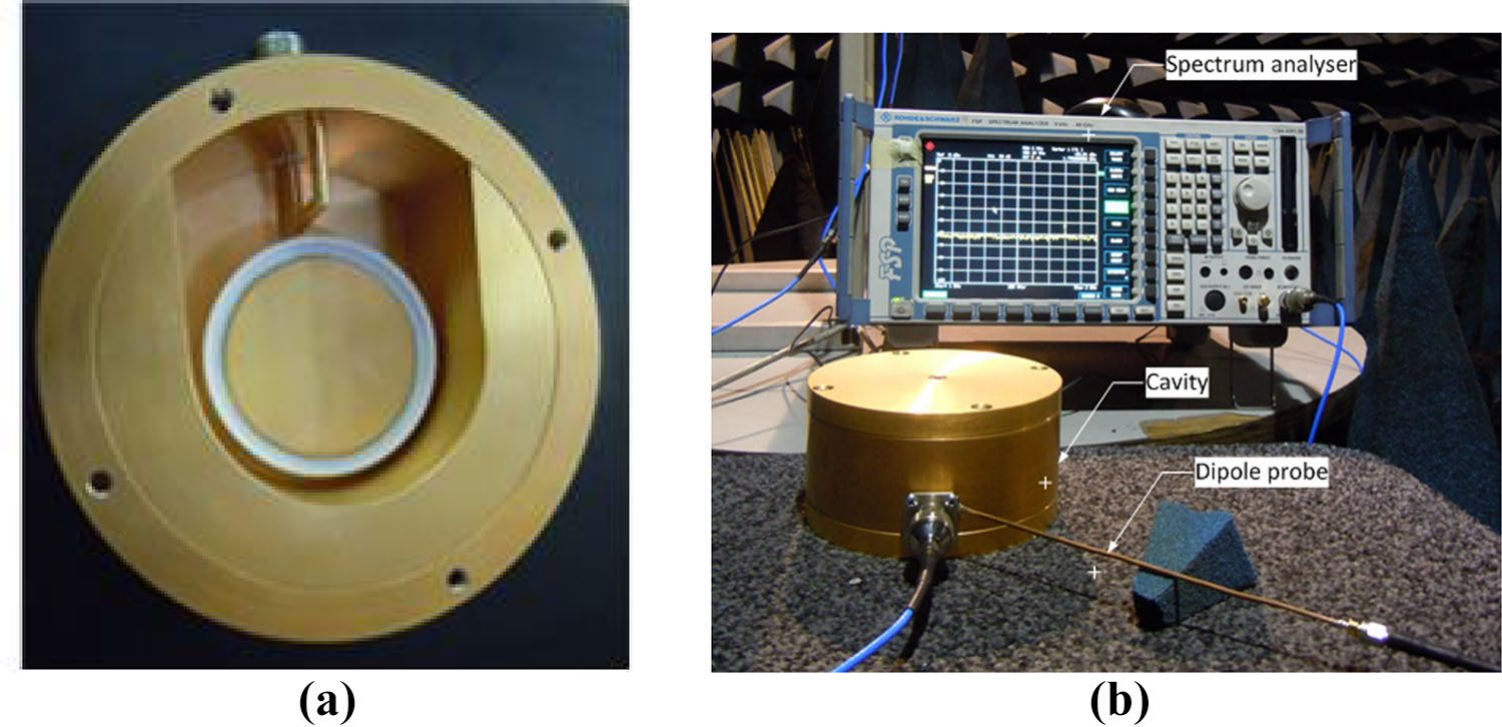
Testing of the fabricated prototype; (**a**) Fabricated exposure system; (**b**) Experimental setup.

The optimised design of the cylindrical re-entrant cavity resonator was fabricated in the Machine Workshop and tested in the Antenna Measurement Laboratory at Queen Mary University of London. The cavity walls are constructed of aluminium and contains the PTFE collar. Brass is used for the two coupling loops. A 50 ohm N-type connector is included to port the excitation signal. The fabricated exposure system is shown in Fig. 10a. To reduce the effects of corrosion and improve the performance, the fabricated cavity was gold-plated through gold electroplating with an effective gold layer thickness of 25 μm (Fig. 10a).

The cavity performance was analysed in both the unloaded and loaded conditions. Since, saline solutions play an important role in many chemical and biological systems, they are a useful tool to establish the working of biological exposure systems. Therefore, the working of the fabricated cavity in loading condition was tested through reflection coefficient measurements carried out using a saline solution (224.5 g/L solution of sodium chloride supplied by APC Pure and deionised water). The petri dish contained 12 ml of the saline solution, similar to the conditions for the simulated cell culture exposure. The cavity was excited using the larger coupling loop in loading condition.

#### Reflection coefficient measurements

The fabricated cavity was first tested by measuring the reflection coefficient ($S_{11}$) in different configurations to confirm the simulation results. A two-port VNA (Hewlett Packard 8720ES-VNA) shown in Fig. 10b was used for the measurements.

**Figure 11:**
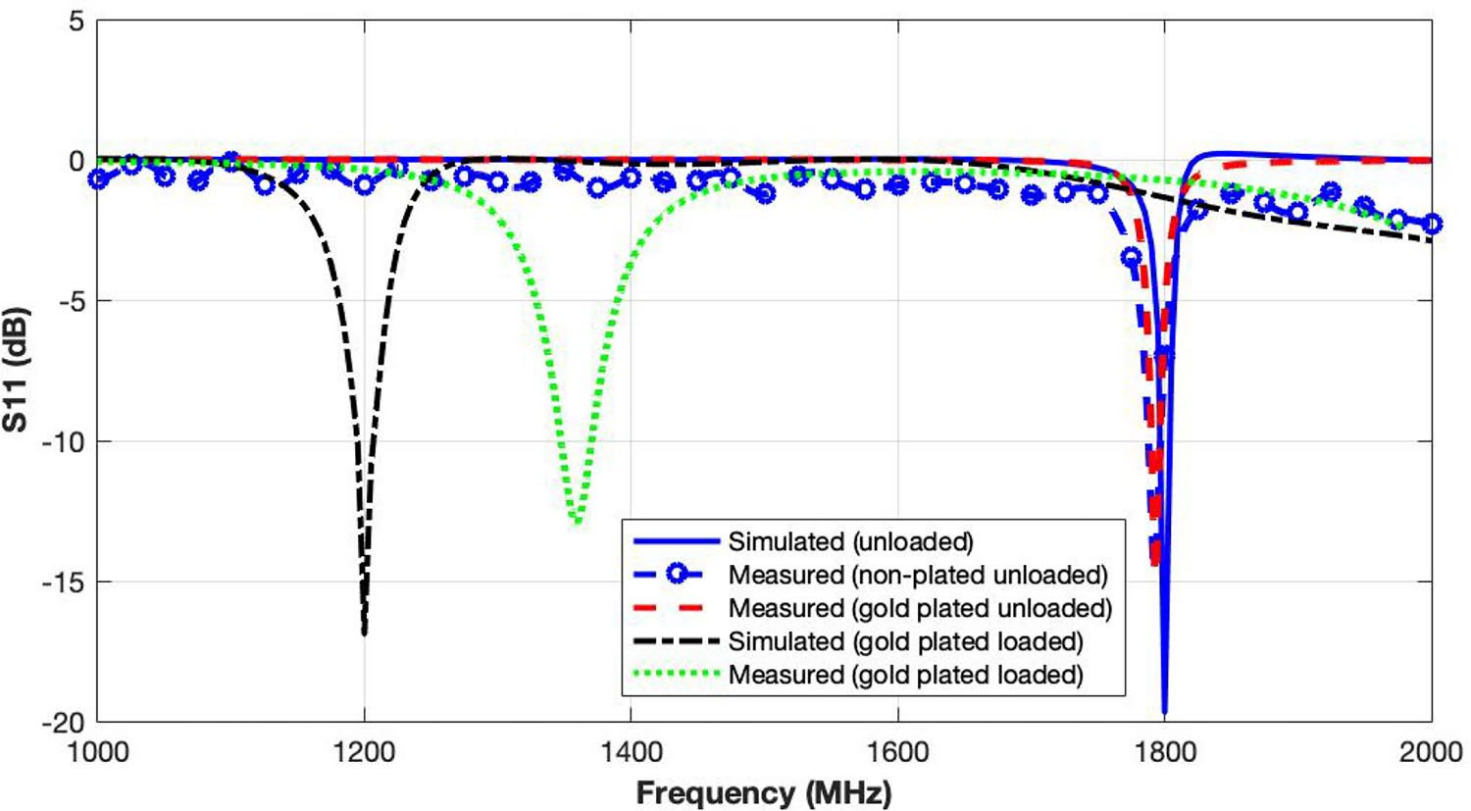
Reflection coefficient measurements of the exposure system comparing the simulated and measured reflection coefficients for non-plated and gold-plated unloaded and loaded (with 12 ml of cell culture) cavity.

The comparison of measured reflection coefficients for non-plated (aluminium) cavity and gold-plated cavity in unloaded conditions with the simulated response is illustrated in Fig. 11. The three results agree very well. It is observed that gold-plating has improved the performance, making the reflection coefficient stable and removing the ripples. The simulated and measured results of the gold-plated cavity in unloaded and loaded with petri dish containing the saline solution also exhibit a very good agreement. The unloaded cavity operates well at 1800 MHz in simulation and 1790 MHz in measurement. The loaded cavity also performs well in terms of containing the detuning caused by the saline solution replicating cell culture. The resonance frequency is noted to be 1325 MHz in simulation versus 1360 MHz in measurement, close to the centre frequency of 1500 MHz in the 1000-2000 MHz frequency band of interest.

#### Power leakage test

As the exposure system has very high input power levels, power leakage can cause severe damage to the power amplifier, incubator and other modules. To ensure safety of the system, the proposed cavity was tested for electromagnetic leakages. A signal generator, spectrum analyser and a dipole probe were used for this purpose. A 25 dBm CW (continuous wave) generated by the signal generator was fed to the cavity. The probe was used to monitor any changes in the spectrum at 1790 MHz to observe power leakages at different positions, especially near the feeding point. The cavity was placed inside an anechoic chamber to mitigate electromagnetic interference.

**Figure 12:**
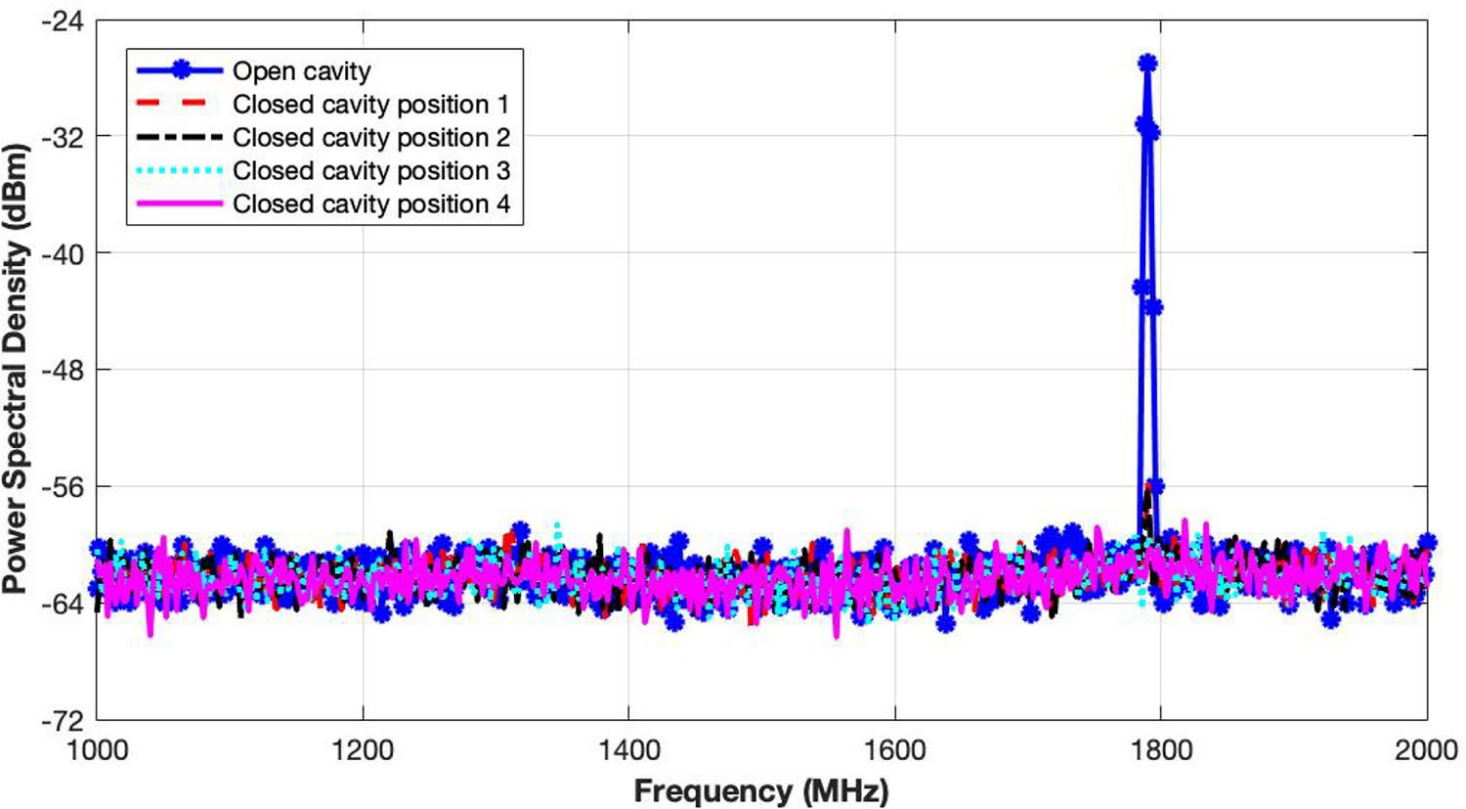
Power leakage test measurements of the gold-plated exposure system through observation of power spectral densities at different points on the cavity.

The spectrum response presented in Fig. 12 shows that the probe picks up a high signal at 1790 MHz near the excitation loop for the open cavity. However, for the closed cavity, no signal above the noise floor is noted. The strongest signal in this configuration is of −56 dBm level. It clearly indicates that the exposure system has no significant level of power leakage. The fabricated exposure system is therefore, ready for the biological studies.

### Ethics approval

This study was conducted in accordance with the International Ethical Guidelines for Epidemiological Studies. This study was exempt from the application of these guidelines because no human subject was used in the investigations.

## Results

The RF exposure system based around the resonant cavity has been simulated, prototyped and tested. The results show that the system achieves the required set of specifications for high pulsed field exposure at electric fields >100 kV/m. This section details the experimental use of the system to study the exposure of cell cultures.

### Experimental exposure setup

The resonant cavity was driven by 200 W (53 dBm) of RF energy. The signal was generated by an Agilent E4432B signal generator and is split using a 3 dB splitter. One half was monitored by a Tektronix DPO7254 oscilloscope, and the second half was fed via an Amplifier Research (AR) RF power amplifier 250W1000C into the input of the Varian VZL-6943G5 TWT amplifier. The output of the TWT amplifier was then fed through an Amplifier Research DC7144A directional coupler and Succofeed transmission line to the resonant cavity. The directional coupler protected the output stage of the power amplifier from reflection and also allowed an Agilent E4417A power meter to monitor the forward and reverse power between the amplifier and the resonant cavity. The pulsing was provided by the Agilent 8118A pulse generator, the output of which was monitored by the oscilloscope and fed into the signal generator to control the output signal generator. The resonant cavity was placed inside an incubator to control the temperature at 37 °C. The fixed exposure conditions included 1195 MHz signal at 53 dBm peak power with a pulse width of 550 nS. The duration of the exposure and the duty cycle (interpulse interval) were altered to control the exposure level.

#### Cell dosimetry

GG0257 cells (European Collection of Cell Cultures, Public Health England, UK) were cultured in RPMI 1640 medium (Sigma). The cells were counted and re-suspended in 164 ml fresh medium contained in a 150 cm$^2$ cell culture flask (vented lid). The flask was incubated at 37°C in an atmosphere of 5% CO$_2$ for 30 min to enable equilibration of CO$_2$. 26 ml of cells were placed in universal tubes, lids sealed tight to maintain the CO$_2$ balance in the medium and the tubes were then transferred to the RF exposure facility and placed in a 37 °C incubator. 12 ml of cells were placed in the petri dish of the RF resonant cavity and 12 ml of cells were placed in a second resonant cavity and sham exposed. The cell density was 3-8 $\times$ 10$^6$ cells and the cells were utilized at passage 8–12. The cells were RF or sham exposed for 6 or 18 min and a range of duty cycles were tested. Post-exposure, the cells were placed in universal tubes for transportation back to the laboratory. The samples were then transferred into petri dishes and incubated at 37 °C in an atmosphere of 5% CO$_2$ for 3 h. Cell viability (as described below) was determined immediately after this 3 h incubation.

#### Cell viability study

The 12 ml cell samples RF/sham exposed were centrifuged (300 $\times$ g; 5 min; 5 °C), washed with ice cold phosphate buffered saline (PBS) and stained with annexin V-FITC and propidium iodide (PI) using the Immunotech Annexin V-FITC kit (Beckman Coulter) according to the manufacturer’s instructions. The prepared samples were analysed using a BD FACSCanto II flow cytometer with FACsDiva version 6 software, to show populations of viable cells (unstained), apoptotic cells (annexin V-FITC stained) and necrotic cells (PI and Annexin V-FITC stained).

#### Temperature measurements

Temperature measurements were undertaken with an Opsens fibre optic thermometer system comprising a fibre optic probe and measurement system. Temperature was measured pre- and post-RF exposure, but it was not possible to record during the exposure as the resonant cavity is a closed system. Pre- or immediately post- exposure, a probe was placed into the medium, the lid of the resonant cavity was then replaced, and this held the probe in place. The incubator door was shut and the temperature change recorded by the fibre optic unit. For the measurements of cooling post-exposure, a range of exposure times and duty cycles were used.

To give an indication of the temperature of the medium in the petri dish during RF exposures, 5-point temperature indication discs (Part No: SC5 / Type 01 / C) made by Omega (Manchester, UK) were used. The indication disc was attached to the centre of the petri dish at a fixed location for each exposure; a range of duty cycles and exposure durations were investigated. Each circle on the disc indicates a specified temperature and, if this temperature is achieved during exposure, the disc changes colour permanently.

### Results and analysis

**Figure 13:**
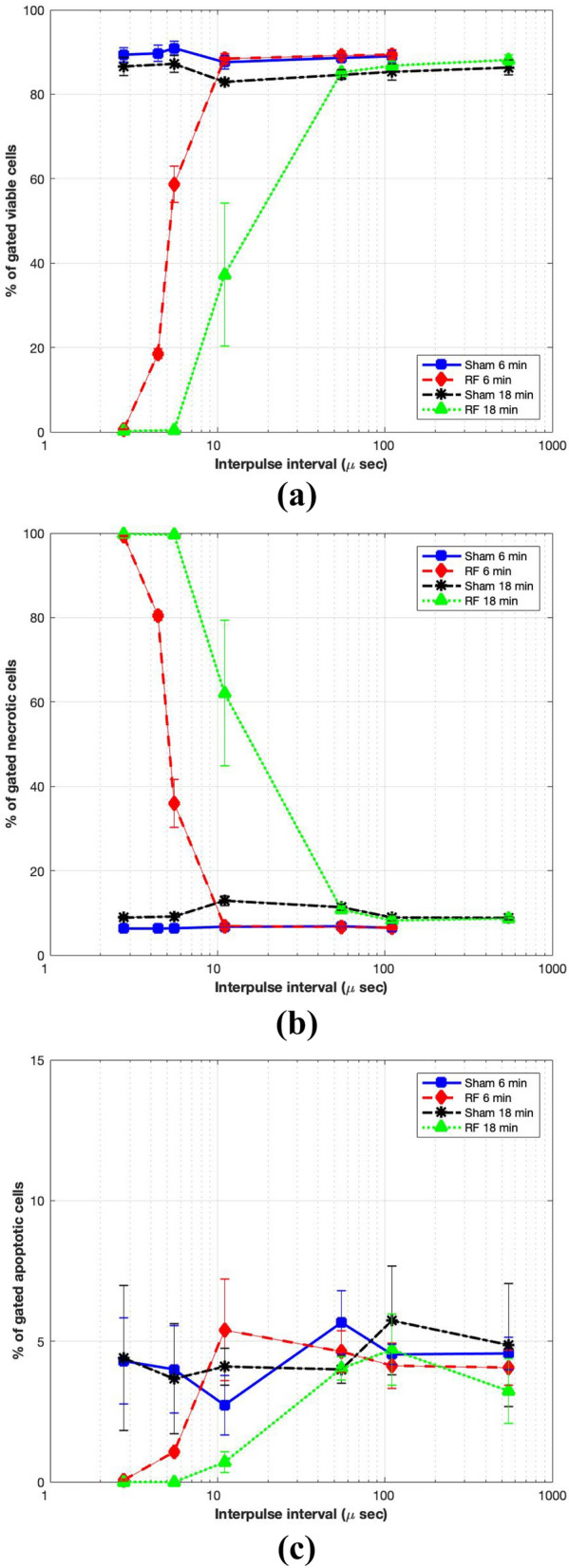
Effect of 6/18 min exposures to interpulse intervals of 2.75, 4.4, 5.5, 11, 55 and 110 μs (RF 1.2 GHz, 550 ns pulse) on cell viability (data shown represent the mean $+/-$ SEM (n=3)); (**a**) % viable cells; (**b**) % necrotic cells; (**c**) % apoptotic cells.

Measurements of cell viability were carried out using the Annexin V-FITC and PI method at 3 hours after a 6 or 18 min RF/Sham exposure method and the results of 3 independent experiments at each duration of exposure are shown in Fig. 13. The experiments were carried out using a constant peak field and increasing the duty cycle (by decreasing the interpulse interval). As the length of exposure increased, the interpulse interval which had an effect on viability also increased. For a 6 min exposure duration, interpulse intervals of 5.5 μs or less resulted in reduced cell viability, with no viable cells present when the interpulse interval was 2.75 μs. For the longer 18 min exposure duration, the reduction in cell viability was observed at longer interpulse intervals, 11 μs or less. No change in viability was observed at interpulse intervals of 11 μs (6 min duration) or 55 μs (18 min duration). The results also demonstrate that the change in viability was due to necrosis rather than apoptosis.

**Figure 14:**
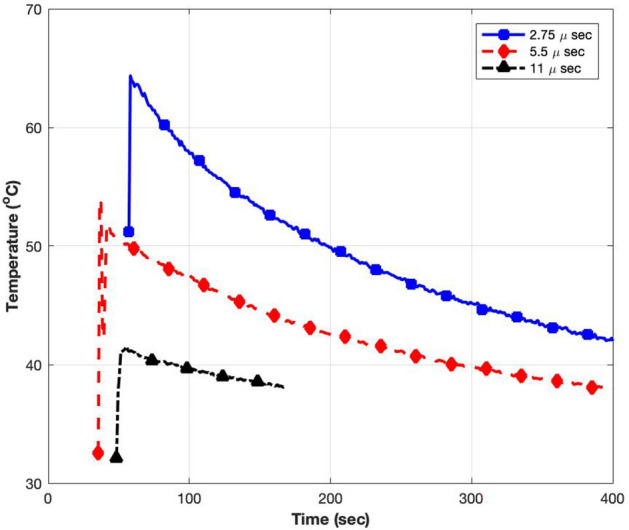
Measurements of media temperature immediately after 18 min exposures, at interpulse intervals of 2.75, 5.5 and 11 μs.

The ultimate aim of this work is to investigate the possible relationship between peak field intensity and biological effects. In order to do this, it is first necessary to define the threshold for heating-induced effects. Measurements of the culture medium temperature were carried out by placing a fibre optic sensor (n = 3) into the petri dish as soon as possible (approximately 10 s) after exposure. Figure 14 shows representative temperature recordings after 18 min exposures at interpulse intervals of 2.75, 5.5 and 11 μs. Temperatures greater than 60 °C were achieved with an interpulse interval of 2.75 μs. The peak recorded temperatures are noted to be 65 °C, 55 °C, and 42.5 °C, respectively, followed by cooling curves. Increasing the interpulse interval resulted in less heating, and no temperature rise could be detected for an interpulse interval of 55 μs (data not shown).

**Figure 15:**
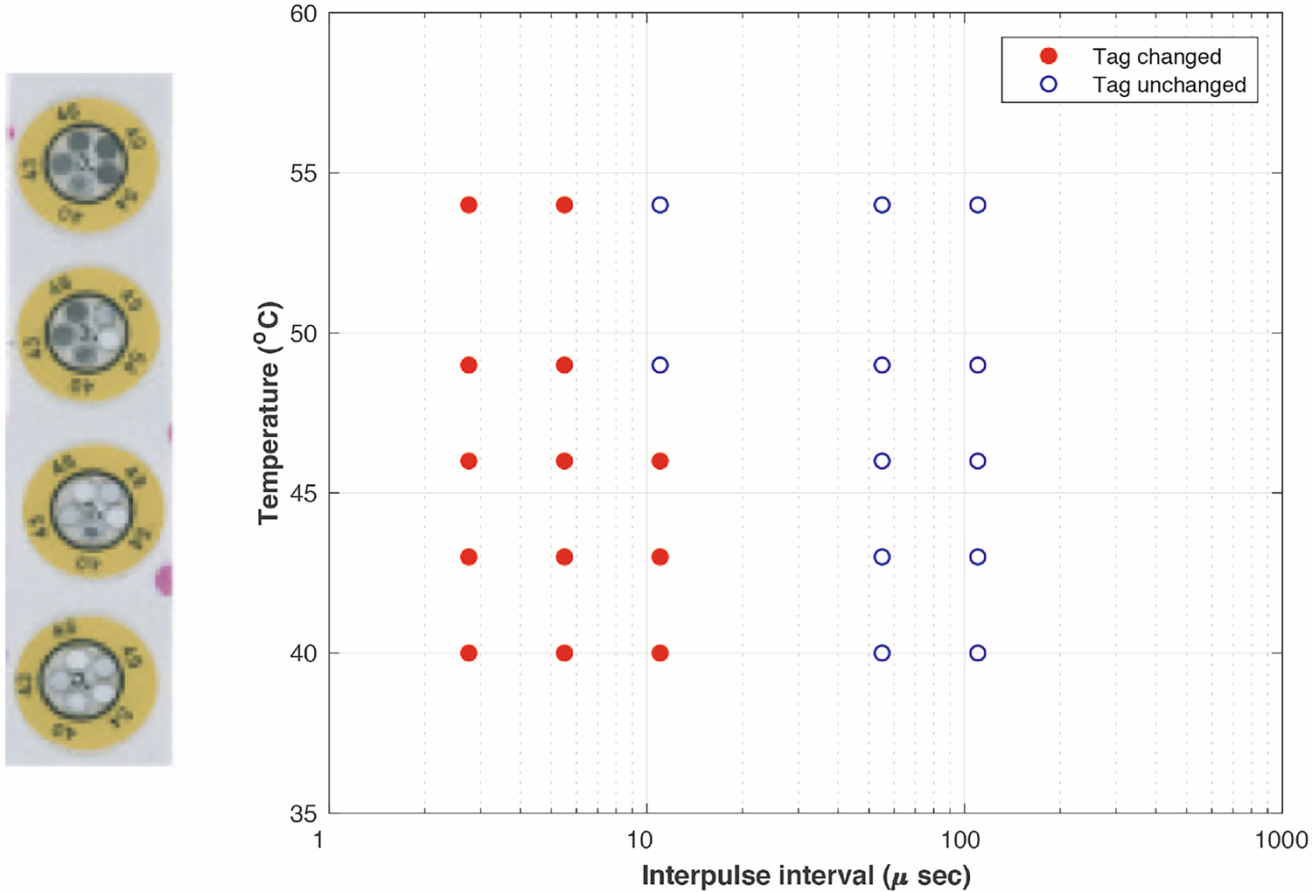
Response of temperature indicator discs to a range of interpulse intervals for a duration of 18 min (RF 1.2 GHz, 550 ns pulse), (n = 3).

An independent indication of the temperature during 18 min exposures was achieved using temperature indicator discs. The results of 3 independent experiments at 18 min exposure duration are shown in Fig. 15. It can be observed that interpulse intervals of 11 μs raised the temperature in the chamber to at least 46 °C (but lower than 50 °C). Interpulse intervals of 2.75 and 5.5 μs raised the temperature above 54 °C whereas interpulse intervals of 55 and 110 μs did not raise the temperature in the chamber above 40 °C.

Based on the observations from Fig. 13, it can be deduced that a temperature range of 43–46 °C is required to initiate a decrease in cell viability in the resonant cavity chamber. The cooling curves shown in Fig. 14 and temperature indication disc data given in Fig. 15 further strengthen this understanding.

Additional work was carried out on temperature indicator discs for 18 min exposure duration. Interpulse intervals of 11 μs raised the temperature in the chamber to 46 °C, however, interpulse intervals of 55 μs did not raise the temperature in the chamber above 40 °C. A typical in-vitro heat shock exposure involves heating mammalian cells to 42-45 °C for 20-60 min and then reverting them to normothermic temperatures (37 °C)^58^. The 18 min exposure to 11 μs interpulse interval produced similar conditions to a heat shock exposure and resulted in less than 40% cell viability (Fig. 13).

Exposing cells to RF for 18 min (Fig. 13) demonstrated that cell viability started to reduce at interpulse intervals of less than 55 μs. These results suggest that the heating threshold is between 55 and 11 μs interpulse intervals. Temperatures measured with temperature indicator discs during RF exposures with interpulse intervals of 55 and 11 μs (Fig. 15) were up to 40 °C and up to 46 °C, respectively. Work previously carried out by the team demonstrated that human T cells heated to 43 °C were damaged but mechanisms were upregulated to limit and/or reverse the damage. However, after heating to 46 °C the cells appeared to have accumulated too much damage and cell death occurred in much greater numbers^59,60^. The previous study supports our current findings. To further investigate the heating threshold, initial experiments suggest that interpulse intervals of 27.5 μs for 18 min exposure result in no viable cells, and interpulse interval of 41.25 μs resulted in 23% cell viability after exposure. Further work would produce a full data set for interpulse intervals of 27.5 and 41.25 μs, including completing the cell viability study and assessing the temperature within the RF exposure chamber during exposure with the temperature indicator discs. The results of this study suggest that the threshold for heating-induced cell death is between 41.25 and 55 μs interpulse interval, with a constant peak field and 18 min exposures.

## Discussion

Resonant cavity systems have been used previously for applications such as in-vivo tooth dosimetry^44^, the enzymatic homogeneous hydrolysis of sucrose^45^, dielectric measurements of common solvents^46^ and for electron paramagnetic resonance spectroscopy^47^. They have also been used for exposing biological preparations to RF fields^33,39–42^. The cavity described in this study compares favourably with these published systems, with a Q factor of 5920 in unloaded conditions, compared to 3000 in^40^, 6500 in^44^ and 2073 in^47^. With an input power of 200 W, the cavity was able to achieve a uniform electric field strength of 125 kV/m in unloaded conditions, higher than that reported for some other systems, e.g. 2 kV/m^41,45^ or 10 kV/m^33^. When loaded with a petri dish containing cell culture medium, although the Q factor fell to 57, the cavity was able to generate a field strength of 63 kV/m, which compares favourably with the 30 kV/m reported by Hamzah *et al.* in a cavity loaded with a methanol sample^46^.

Experiments with cell cultures showed that the cavity was able to cause changes in cell viability associated with heating to temperatures of 46 °C. Cell death occurred by necrosis was observed at a lower duty cycle for longer duration exposures, consistent with it being due to temperature rise. This was confirmed by temperature measurements immediately after exposure and by temperature indicator discs attached to the petri dishes during exposure. These showed good agreement and suggested that a temperature of approximately 46 °C was required to induce cell death under these exposure conditions.

Although there was a delay between the end of exposure and the commencement of temperature recording with the fibre optic probe, the recordings showed smooth cooling curves which made it possible to extrapolate back to the time exposure ended. The temperature indicator discs had a much cruder resolution (46 °C) but, since they could be included in the exposure, they provided a useful confirmation of the Opsens measurements.

These experiments have characterised the exposure conditions and the temperature threshold for cell death induced by heating, and have shown that this effect can be avoided by reducing the duty cycle (increasing the interpulse interval) whilst maintaining the same peak electric field strength in the pulses. Future study will utilise longer interpulse intervals to search for effects which are related to peak field strength rather than heating due to energy absorption. In order to do this, it will be necessary to distinguish temperature-dependent nonlethal effects. There are likely to be changes in gene expression, for example, at lower levels of heating which are compensated for by the cell and do not result in cell death.

The present study used a human lymphoid cell line (GG0257) for ease of culture and consistency between plates; however, primary cell cultures might be expected to be more susceptible to environmental stressors and these will be used in future experiments to search for peak field effects. Comparison between the cell line and primary cells may also be useful.

Although, the unloaded cavity was able to achieve field strengths greater than the recommended exposure limit of 100 kV/m in the IEEE 2005 standard, under loaded conditions the maximum electric field strength fell to 63 kV/m. Taking into account the coupling losses of an external field into the human body, however, a field strength of 63 kV/m in the cell culture is likely to represent what would be achieved in many tissues by a much higher external field intensity.

## Conclusion

A detailed study on a resonant cavity-based system for human cell exposure to very high electric fields has been presented. The exposure system is designed numerically and tested experimentally both in unloaded and loaded configurations with effective impedance matching mechanism to mitigate the detuning and impedance mismatch caused by the loading of human cell culture in a petri dish. The system offers good tuning ability, high levels of electric field and SAR uniformity across the cell culture medium. The cavity is also gold-plated to improve the performance. A good agreement between the simulation and measurement of the reflection coefficients has been exhibited by the system with operation in 1000–2000 MHz range and an input of 200 W. Detailed measurements on GG0257 cells using the exposure system were then carried out to verify the working of the developed system. This study has also investigated effects of RF on cell viability and has provided valuable information to enable pulse repetition rates to be selected which are sufficiently low to avoid heat-induced changes. The results show that the designed system is potentially a good candidate to analyse the cell culture changes at the recommended exposure limit of 100 kV/m.

## Data Availability

The data generated or analysed during this study are available from the corresponding author on reasonable request.
